# Cerebrovascular Reactivity Assessment with Breath-Hold Functional MRI in Patients with Moyamoya Angiopathy: Which Time Period to Analyze?

**DOI:** 10.3390/diagnostics16060904

**Published:** 2026-03-18

**Authors:** Leonie Zerweck, Uwe Klose, Constantin Roder, Nadia Khan, Philipp T. Meyer, Ulrike Ernemann, Till-Karsten Hauser

**Affiliations:** 1 Department of Diagnostic and Interventional Neuroradiology, University Hospital Tuebingen, 72076 Tuebingen, Germanytill-karsten.hauser@med.uni-tuebingen.de (T.-K.H.); 2 Department of Neurosurgery, University Hospital Tuebingen, 72016 Tuebingen, Germany; 3 Moyamoya Center, University Children’s Hospital Zurich, 8008 Zurich, Switzerland; 4 Department of Nuclear Medicine, Medical Center—University of Freiburg, Faculty of Medicine, University of Freiburg, 79085 Freiburg, Germany

**Keywords:** Moyamoya Angiopathy, cerebrovascular reactivity, breath-hold functional MRI, [^15^O]water PET

## Abstract

**Background/Objectives**: Quantifying cerebrovascular reactivity (CVR) is essential for stroke risk assessment in patients with Moyamoya Angiopathy (MMA). Breath-hold functional MRI (bh-fMRI) is an easily implementable method to assess CVR. Determining the optimal time period of the BOLD signal for analyzing the best bh-fMRI data quality remains an open question. **Methods**: A retrospective analysis of 46 bh-fMRI data sets of MMA patients was conducted. The percentage BOLD signal changes were evaluated at different time periods (time point of the maximum cerebellar signal peak (TP_cereb. max_) ± 0 s, TP_cereb. max_ ± 1 s, TP_cereb. max_ ± 2 s, TP_cereb. max_ ± 3 s, TP_cereb. max_ ± 4 s, TP_cereb. max_ ± 5 s). The agreement between the bh-fMRI maps and [^15^O]water PET maps was independently and consensually rated on a 4-point Likert scale (1 = poor, 2 = moderate, 3 = good, 4 = excellent) and compared with the Friedman test. The inter-rater agreement was calculated separately for each time period using quadratic weighted Cohen’s kappa κ_w_. **Results**: The selected time period had a significant impact on the agreement between bh-fMRI and [^15^O]water PET (χ^2^(5) = 79.448, *p* < 0.001, W = 0.345). Short time periods of TP_cereb.max_ ± 0 s or TP_cereb.max_ ±1 s demonstrated the highest level of concordance between bh-fMRI and [^15^O]water PET (median = 3.5 for TP_cereb.max_ ± 0 s; median = 3 for TP_cereb.max_ ± 1 s, modus = 4 in both cases). The agreement between bh-fMRI and [^15^O]water PET was significantly higher when evaluating time periods of TP_cereb.max_ ± 0 s than when evaluating all time periods ≥ TP_cereb. max_ ± 2 s. The inter-rater agreement was almost perfect for all time periods except one (TP_cereb. max_ ± 1 s). **Conclusions**: Short time periods should be selected when evaluating CVR with bh-fMRI, as this study suggests a high level of validity in comparison to [^15^O]water PET.

## 1. Introduction

Moyamoya Angiopathy (MMA) is a progressive cerebrovascular disease characterized by stenoses of mainly the anterior circulation, while the posterior circulation is often spared, especially in early stages of the angiopathy [1]. Neuroimaging, including hemodynamic assessment, should be conducted regularly to determine the risk of stroke [2,3,4,5,6,7,8,9,10]. This assessment is crucial in establishing whether revascularization surgery is required to prevent ischemic events [2,3,4,5,6,7,8,11]. The diagnostic gold standard to assess the cerebral perfusion reserve capacity (CPR) is acetazolamide (ACZ)-triggered [^15^O]water PET [8,12,13]. Evaluating cerebrovascular reactivity (CVR) using hypercapnia-triggered functional MRI (fMRI) appears to be a surrogate parameter for CPR and offers wider accessibility [2,3,4,12,13]. CVR is understood to be the local cerebral blood flow change in response to a vasoactive stimulus [3,5,8,12,14,15,16,17,18,19]. The most common vasodilatory stimulus is hypercapnia, which can be achieved through breath-hold periods or by the inhalation of CO_2_ [2,3,4,5,8,12,14,15,16,17,18,20]. Hypercapnia leads to increased cerebral blood flow, altering the ratio of paramagnetic deoxyhemoglobin to diamagnetic oxyhemoglobin and inducing a Blood-Oxygenation-Level-Dependent (BOLD) signal peak in brain parenchyma with preserved CPR [2,8,14,16]. If CPR is reduced, however, only small or even negative BOLD signal peaks are expected [2,6,8,21]. Negative BOLD signal peaks are known as “steal phenomenon” and are caused by the increased cerebral perfusion in surrounding parenchyma, which is “stolen” from the vascular territory with depleted CPR [2,4,8,16,21,22,23].

Short breath-hold periods have been shown to induce BOLD signal peaks with a full width at half maximum (FWHM) of approximately 15 s [14]. The BOLD signal response is expected with temporal delay to the vasodilatory stimulus [2,8,24,25,26]. It should be noted that these delays can differ regionally, e.g., due to vessel stenoses or blood flow via collateral vessels or between white and grey matter [8,16,27,28,29]. Due to regional temporal delays in the breath-hold-triggered BOLD response, some studies have evaluated the BOLD response over a period of time rather than at a single point, where the largest BOLD signal peak could be missed [4,8,14]. A recent study showed that voxel-wise delay correction improves the agreement between bh-fMRI and the diagnostic standard [^15^O]water PET [8].

This raises the following question: When implementing a voxel-wise delay correction, is it necessary to evaluate extended time periods of the BOLD response to achieve stable results, or would shorter time periods, or even individual timepoints, be more effective in revealing regional CVR differences?

The objective of this study was therefore to determine the BOLD signal changes for different time periods and to validate the bh-fMRI data in comparison to the diagnostic standard [^15^O]water PET in MMA patients.

## 2. Materials and Methods

### 2.1. Study Design

A retrospective evaluation of bh-fMRI and [^15^O]water PET data of MMA patients was performed. The study was approved by the institutional ethics committee.

### 2.2. Patients

The inclusion criteria were as follows: Angiographically diagnosed MMA and the availability of bh-fMRI and [^15^O]water PET data, acquired no more than six months apart and with no revascularization performed between the scans. Both bh-fMRI and [^15^O]water PET data were acquired during routine clinical scans. Exclusion criteria comprised known non-vascular cerebral diseases, acute stroke and intracranial hemorrhage. In order to avoid any potential bias, only the first data set was used in cases where multiple data sets were available for a given patient. A power calculation revealed a total sample size of at least 44 patients.

### 2.3. MRI Data Acquisition and Preprocessing

MR imaging was performed on a 3 T MR Scanner (Magnetom Skyra, Siemens, Erlangen, Germany) by use of a 20-channel head coil. The routine MRI protocol included, among others, a bh-fMRI and a dynamic susceptibility contrast (DSC) MR perfusion sequence. The DSC MR sequence served to process the bh-fMRI data.

DSC MRI data were dynamically acquired using an echo-planar imaging (EPI) sequence with the following parameters: TR = 2340 ms, TE = 30 ms, matrix 128 × 128, slice thickness = 5 mm, 30 slices, FOV = 218 mm, resolution = 1.7 × 1.7 × 5.0 mm, 60 measurements, TA = 1:58 min. A bolus of contrast agent (Gadobutrol, 0.15 mmol/kg of body weight) was administered intravenously at a rate of 3 mL/s and subsequent flushed with 25 mL saline.

The fMRI scan was performed using a T2*-weighted EPI sequence with the following parameters: TR = 3000 ms, TE = 36 ms, matrix 96 × 96, slice thickness = 3 mm, 34 slices in interleaved ascending order, FOV = 245 mm, resolution = 2.6 × 2.6 × 3.0, 181 measurements, TA = 8:10 min.

The breath-hold protocol was initiated with a 60 s period of normal breathing, followed by seven periods of 9 s end-expiratory breath-holding and 60 s of regular breathing, as previously described [8]. The instructions were displayed using a wall-mounted screen and a mirror mounted to the head coil. The breath-hold stimuli were synchronized with the scanner and delivered with Presentation V20.1 (Neurobehavioral Systems, Berkeley, CA, USA).

All DSC MRI and bh-fMRI data were pre-processed using Statistical Parameter Mapping (SPM12) running on MATLAB (R2018b (The MathWorks, Inc., Natick, MA, USA). First, conversion of the DICOM images into NIfTI format was performed. Second, the images were slice-timing corrected and realigned to compensate for head movement, after which these were normalized to Montreal Neurological Institute (MNI) space. Further data processing was performed using in-house scripts written in MATLAB [2,3,4,5,8,14,30,31]. The DSC MRI data were used to calculate voxel-wise bolus arrival times, which were then used to correct the bh-fMRI data [8]. Next, patient compliance in performing the breath-hold paradigm was verified. As initially described by Hauser et al., the cerebellar signal time course was temporally averaged over the seven breath-hold periods [4]. The mean cerebellar signal time course was used as a reference as proposed in previous studies [4,32]. A physiological BOLD response is expected in patients with MMA, because it mostly affects the anterior circulation, and the cerebellum, which is supplied by the posterior circulation, is much less frequently affected [2,3,4,7]. Periods with no cerebellar BOLD signal peak were excluded from the further evaluation [4,8,31]. The remaining periods were averaged [4,8]. Next, the BOLD signal time-course of each voxel was detrended in order to eliminate linear signal trends. The BOLD signal time-course was time-shifted voxel-wise based on the calculated bolus arrival times to correct for hemodynamic delays [8]. To conclude, we calculated the percentage BOLD signal change relative to baseline for each voxel. This was performed by calculating the percentage BOLD signal change at the time point of the maximum cerebellar signal peak (TP_cereb. max_) ± 0 s, TP_cereb. max_ ± 1 s, TP_cereb. max_ ± 2 s, TP_cereb. max_ ± 3 s, TP_cereb. max_ ± 4 s and TP_cereb. max_ ± 5 s. These time periods of up to 10 s were selected based on a previous study, which described BOLD signal peaks with a FWHM of approximately 15 s following breath-hold periods of 9 s [14], because we intended to cover the main peak with our analysis while avoiding other signal changes, such as so-called recovery peaks that might occur after the steal phenomenon [2]. Next, 120 predefined anatomical volumes of interest (VOIs) were used and the mean percentage BOLD signal change values in these VOIs were color-coded with standardized color scales and overlaid on the normalized standard brains.

### 2.4. [^15^O]Water PET Data Acquisition and Preprocessing

PET imaging was performed using a Philips Vereos digital PET/CT system. Two 4-min PET scans were acquired at baseline and following stimulation with ACZ (5-min infusion (10 mL) of a standard dose of 1000 mg). The bolus injection accounted for 291.2 ± 23.7 MBq [^15^O]water per scan. The time interval between successive scans was 10 min. The ACT infusion commenced immediately after completion of the second baseline scan, while the first post-ACZ scan started 10 min after the beginning of the infusion. Data preprocessing was performed as previously outlined [2,3,8]. CPR maps were generated by calculating the voxel-wise percentage signal change between the mean baseline and post-ACZ maps, following a preciously described approach [33]. Images were obtained by integrating the dynamic PET data over a 60-s interval starting at tracer arrival in the individual patient’s brain [34]. CRP maps were visualized in 32 transaxial slices using a standardized “rainbow” color scale. Display limits were set symmetrically around zero (± [mean value + 2 standard deviations of CPR in cerebellar and PCA territories]). The zero crossing indicating steal phenomenon was indicated by a black bin within the 256-bin scale.

### 2.5. Data Evaluation and Statistical Analysis

The agreement between the individual color-coded bh-fMRI maps and [^15^O]water PET maps of each patient was rated independently and consensually by two neuroradiologists (rater A with 23 years of experience, rater B with 3 years of experience) using a 4-point Likert scale (1 = poor agreement, 2 = moderate agreement, 3 = good agreement, 4 = excellent agreement). All maps were presented on a designated workstation.

The inter-rater agreement was calculated separately for all data and for the individual time periods using quadratic weighted Cohen’s kappa κ_w_. Inter-rater agreement was interpreted using the following κ_w_ ranges: slight 0–0.20; fair 0.21–0.40; moderate 0.41–0.60; substantial 0.61–0.80 and almost perfect 0.81–1.00 [35].

Modus and median were calculated for all ratings. The Friedman test with Dunn post-hoc tests was used to compare the consensus ratings of the agreement between the bh-fMRI and the [^15^O]water PET maps. *p* values below 0.05 were considered significant, and the false discovery rate (FDR) method by Benjamin and Hochberg was used to correct for multiple comparisons. The Kendall’s W effect size was determined for the Friedman test and the effect size r was assessed for the post-hoc tests. Kendall’s W was interpreted using the following ranges: small effect > 0.1; medium effect > 0.3; large effect > 0.5 and the effect size r was understood as follows: small effect > 0.1; medium effect > 0.3; large effect > 0.5 [36].

Due to the considerable time period of six months between the MRI and the [^15^O]water PET scan, as specified in the inclusion criteria, a supplementary subgroup analysis was conducted for the inter-scan time periods of 0–3 months and 3–6 months. This analysis involved the comparison of the consensus ratings of the agreement between the bh-fMRI and the [^15^O]water PET maps.

## 3. Results

In total, 47 data sets fulfilled the inclusion criteria. Forty-five data sets of patients without prior surgery and two data sets of patients with unilateral superficial temporal artery (STA)–middle cerebral artery (MCA) bypass were included. Data of one patient were excluded from further analysis due to incorrect execution of the breath-hold paradigm. The summary of patient data is shown in Table 1.

### 3.1. Influence of the Evaluated Time Periods of bh-fMRI on Agreement with [^15^O]Water PET

**Figure 1:**
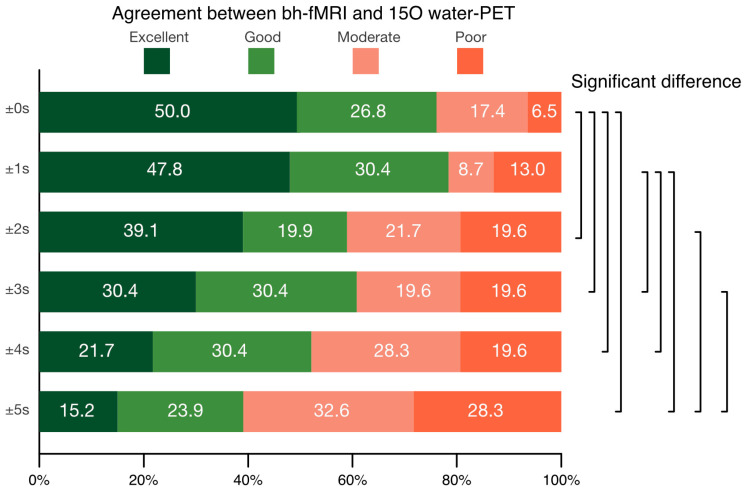
Consensus rating of the agreement between the breath-hold fMRI (bh-fMRI) and the [^15^O]water PET maps when evaluating bh-fMRI time periods of ±0 s, ±1 s, ±2 s, ±3 s, ±4 s, ±5 s around the time point of the maximum cerebellar BOLD signal peak. Significant differences are marked on the right.

The evaluation of different time periods significantly affects the visual agreement between bh-fMRI and [^15^O]water PET with medium effect size (χ^2^(5) = 79.448, *p* < 0.001, W = 0.345) (see Figure 1).

The evaluation of TP_cereb. max_ ± 0 s yielded the highest agreement between bh-fMRI and [^15^O]water PET, rated as good to excellent (consensus median = 3.5, modus = 4). The agreement between bh-fMRI and [^15^O]water PET was significantly higher than that of all other time periods (TP_cereb. max_ ± 0 s vs. TP_cereb. max_ ± 2 s: *p* = 0.040, r = 0.130; TP_cereb. max_ ± 0 s vs. TP_cereb. max_ ± 3 s: *p* = 0.013, r = 0.162; TP_cereb. max_ ± 0 s vs. TP_cereb. max_ ± 4 s: *p* < 0.001, r = 0.218; TP_cereb. max_ ± 0 s vs. TP_cereb. max_ ± 5 s: *p* < 0.001, r = 0.313), except for time period TP_cereb. max_ ± 1 s (*p* = 0.676).

Evaluating the time period TP_cereb. max_ ± 1 s yielded an only slightly and non-significantly lower rating (consensual median = 3, modus = 4) than evaluating TP_cereb. max_ ± 0 s and led to a significantly better rating than the evaluation of the time periods TP_cereb. max_ ± 3 s (*p* = 0.032, r = 0.138), TP_cereb. max_ ± 4 s (*p* = 0.003, r = 0.194) and TP_cereb. max_ ± 5 s (*p* < 0.001, r = 0.289).

The evaluation of the time periods TP_cereb. max_ ± 2 s, TP_cereb. max_ ± 3 s and TP_cereb. max_ ± 4 s revealed good agreement between bh-fMRI and [^15^O]water PET (consensus median = 3; modus = 3 or 4). The time periods TP_cereb. max_ ± 2 s (*p* = 0.019, r = 0.183) and TP_cereb. max_ ± 3 s (*p* = 0.019, r = 0.148) showed significantly higher agreement with [^15^O]water PET than TP_cereb. max_ ± 5 s.

In the subgroup of patients with an interval of 0–3 months between MRI [^15^O]water and [^15^O]water PET scan (*n* = 33), the evaluated time periods also had a significant effect on the agreement between bh-fMRI and [^15^O]water PET with medium effect size (χ^2^(5) = 53.535, *p* < 0.001, W = 0.325). A similar effect was observed in the subgroup of patients with an interval of 3–6 months between the scans (*n* = 13) (χ^2^(5) = 26.547, *p* < 0.001, W = 0.409) (see Supplementary Figure S1). The highest level of agreement between bh-fMRI and [^15^O]water PET was detected at time periods TP_cereb. max_ ± 0 s and TP_cereb. max_ ± 1 s (inter-scan interval of 0–3 months: consensus median = 3, modus = 4 in both cases, inter-scan interval of 3–6 months: consensus median = 4, modus = 4 in both cases). As the number of patients in the subgroup analysis decreased, the number of significant differences found in the post-hoc analyses decreased accordingly (see Supplementary Figure S1).

**Figure 2:**
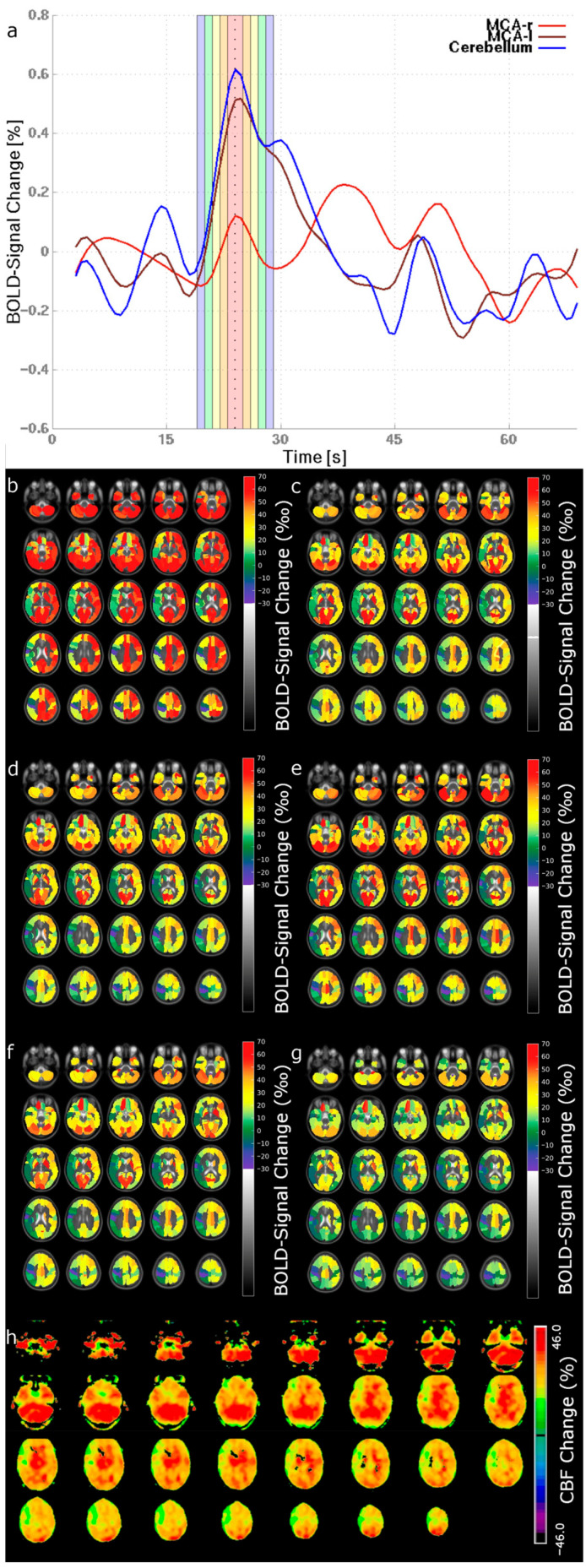
Breath-hold fMRI and [^15^O]water PET data of one patient with unilateral right-side Moyamoya Angiopathy. (**a**) shows exemplary mean BOLD signal time courses of three territories (MCA-r = right middle cerebral artery; MCA-l = left middle cerebral artery; cerebellum). The colored bars indicate the evaluated time periods (dotted line = time point of the maximum cerebellar signal peak (TP_cereb. max_) ± 0 s; red bar = TP_cereb. max_ ± 1 s; orange bar = TP_cereb. max_ ± 2 s; yellow bar = TP_cereb. max_ ± 3 s; green bar = TP_cereb. max_ ± 4 s; blue bar = TP_cereb. max_ ± 5 s). (**b**–**g**) show the breath-hold fMRI maps for the individual time periods (**b** = TP_cereb. max_ ± 0; **c** = TP_cereb. max_ ± 1 s; **d** = TP_cereb. max_ ± 2 s; **e** = TP_cereb. max_ ± 3 s; **f** = TP_cereb. max_ ± 4 s, **g** = TP_cereb. max_ ± 5 s) and (**h**) illustrates the maps of the reference method [^15^O]water PET.

Figure 2 shows exemplary maps of a patient with unilateral MMA. This example illustrates why larger evaluated time periods can lead to a lower level of agreement between bh-fMRI and [^15^O]water PET: As illustrated, the mean BOLD values in the right MCA territory are positive for short time periods, while they become increasingly negative for larger time periods. This is evident in both the signal time courses and the bh-fMRI maps. At larger evaluated time periods, steal phenomena can be observed in certain brain regions. In contrast, [^15^O]water PET reveals lower CBF changes on the right than on the left side, but the right hemisphere does not exhibit steal phenomena. Therefore, bh-fMRI maps with shorter evaluated time periods correspond better with the [^15^O]water PET maps than those with larger time periods. In general, BOLD signal values tend to be positive when evaluating a single time point or a short time period, while longer time periods can lead to smaller and sometimes negative BOLD signal changes with an apparent steal phenomenon. This usually results in a lower visual concordance between bh-fMRI and the [^15^O]water PET diagnostic standard.

### 3.2. Inter-Rater Agreement

The inter-rater reliability across all evaluated time periods was almost perfect (κw = 0.828, 95% confidence interval (CI) = 0.785–0.871, *p* < 0.001). The inter-rater reliability across individual time periods was almost perfect for TP_cereb. max_ ± 0 s (κw = 0.929, 95% CI = 0.872–0.987, *p* < 0.001), TP_cereb. max_ ± 2 s (κw = 0.901, 95% CI = 0.832–0.970, *p* < 0.001), TP_cereb. max_ ± 3 s (κw = 0.914, 95% CI = 0.855–0.973, *p* < 0.001), TP_cereb. max_ ± 4 s (κw = 0.938, 95% CI = 0.889–0.987, *p* < 0.001) and TP_cereb. max_ ± 5 s (κw = 0.922, 95% CI = 0.870–0.975, *p* < 0.001) and substantial for TP_cereb. max_ ± 1 s (κw = 0.803, 95% CI = 0.700–0.906, *p* < 0.001).

## 4. Discussion

### 4.1. Motivation of the Study and Main Findings

CVR assessment is necessary in patients with MMA [2,4,9]. Although the prevalence of MMA is relatively low—up to 10.5 per 100,000 in Japan [37,38]—CVR assessment remains highly relevant because it helps guide decisions regarding bypass surgery [2,3,4,5,6,7,11]. [^15^O]water PET is the diagnostic gold standard for hemodynamic evaluation [8,12,13]. However, bh-fMRI has the advantage of being more easily repeatable due to lower costs and greater availability [2]. Furthermore, CVR assessment is relevant in patients with more prevalent cerebrovascular diseases such as atherosclerotic extracranial and intracranial carotid artery stenosis [17,39,40]. In recent years, CVR has also been increasingly investigated in a variety of other diseases [19,30,31,41,42,43], including small vessel disease [44,45,46,47], neurodegenerative diseases [48,49,50,51,52], traumatic brain injury [53,54], and epilepsy [31] as well as in neurooncology [42,55,56,57,58,59,60]. This also supports the rationale for continued research in CVR assessment and the development of methodical improvements.

The objective of this study was to investigate which time periods should be analyzed when assessing CVR with bh-fMRI.

In order to ensure the appropriate use of bh-fMRI in routine clinical diagnostics, the quality criteria of objectivity, reliability, and validity must be met. While Renger et al. evaluated the reliability of bh-fMRI over different time periods [61], our primary aim was to address the question of validity.

In this study, the validity of bh-fMRI data across different time periods was evaluated by comparing them to the diagnostic standard [^15^O]water PET. The results of this study indicated that single time points (TP_cereb. max_ ± 0 s) or small time periods of TP_cereb. max_ ± 1 s led to significantly greater agreement with the [^15^O]water PET, than time periods of ≥TP_cereb. max_ ± 2 s, suggesting that bh-fMRI has greater validity when evaluating periods of shorter duration. This was evident irrespective of the time interval between the bh-fMRI and the [^15^O]water PET scan. However, it should be mentioned that the effect size of our analysis was medium, indicating variance in patients’ response to the different evaluated time periods.

In our analysis we observed that longer time-periods tend to result in lower CVR values and even steal phenomena, which were not always evident in [^15^O]water PET. On the one hand, it might be possible that [^15^O]water PET fails to capture the steal phenomenon. But on the other hand, the underlying reason could be a mathematical phenomenon in which integrating over longer time periods results in negative values. As [^15^O]water PET is the diagnostic standard in hemodynamic evaluation of patients with MMA [8,12,13], we believe that the negative values are more likely a mathematical artefact than an real steal phenomenon that is not captured with [^15^O]water PET.

We calculated the inter-rater reliability for each time period separately. Our analysis revealed almost perfect inter-rater reliability except for the TP_cereb. max_ ± 1 s time period, for which categorization as almost perfect was narrowly missed (κw = 0.803 instead of 0.81), suggesting a little more volatility in the TP_cereb. max_ ± 1 s time period compared to the other time periods. Overall, the inter-rater reliability indicates a high level of consistency in interpreting bh-fMRI data.

### 4.2. Review of Previous Studies on CVR Experiments in MMA

Renger et al. examined healthy participants and analyzed the test–retest reliability of bh-fMRI at different time intervals. Their findings showed excellent overall reproducibility, but shorter time periods resulted in significantly higher test–retest reliability.

In summary, the results of this study and the findings of Renger et al. suggest that shorter time periods lead to greater validity and test–retest reliability of bh-fMRI, while inter-rater reliability appears to be high for all time periods evaluated. For this reason, short time periods are recommended for clinical bh-fMRI evaluation.

It must be emphasized that different post-processing approaches for bh-fMRI data exist [16,41,62]. Our analysis focused on one method, in which the mean BOLD signal change is calculated over a pre-defined time period [2,3,4,5,8,14,30,31]. Other approaches for hypercapnia-triggered bh-fMRI evaluation rely, e.g., on linear regression analysis or its extension with a general linear model (GLM) [16,46,63,64]. The results of this study cannot be directly transferred to other post-processing approaches. Furthermore, this study focused on patients with MMA, where CVR can be severely compromised. The transferability of the results to other diseases is limited.

In this analysis, voxel-wise time delay correction of the BOLD signal was performed, as proposed in recent studies [8,64,65,66]. This step is particularly important when examining patients with MMA, where regional perfusion delays and consecutive BOLD signal delays are to be expected due to vessel stenosis and collateral circulation [8]. After accounting for these perfusion delays, we selected the time point of the cerebellar BOLD signal peak as the reference time point for further analysis. The cerebellum is usually not affected by the disease because it is supplied by the posterior circulation [7,14]. However, it should be noted that, particularly in later stages of the disease, the posterior circulation may also be affected in MMA [67]. As isolated involvement of the posterior circulation is rare, we still consider the cerebellum to be the best region for comparison in MMA. Delay correction based on DSC MRI allowed us to use a uniform evaluation period for the analyses. Without delay correction, the analysis might not have concluded that short time periods are suitable because it would have been highly probable that the maximum signal response would not have been evaluated.

### 4.3. Limitations

This study has limitations that should be addressed. The most considerable limitation pertained to the comparison between bh-fMRI and [^15^O]water PET, which was based on a subjective rating by two neuroradiologists. A VOI-by-VOI correlation analysis would have been more objective but was not possible due to the retrospective study design and limited raw data availability. The bh-fMRI and [^15^O]water PET maps were evaluated in accordance with our standard clinical routine approaches. For this reason, the calculation of CPR in [^15^O]water PET was performed on a voxel-by-voxel basis, while CVR in the bh-fMRI maps was presented in small anatomical VOIs, resulting in another spatial resolution. This may have had an impact on the rating, as subregional patterns, such as focal areas of steal phenomenon, may have been masked in the bh-fMRI maps due to the averaging of the CVR values while [^15^O]water PET would detect them with more accuracy. However, we chose this approach because we wanted to improve the way we process bh-fMRI scans in clinical routine. All bh-fMRI scans were analyzed in the same 120 predefined VOIs, so we think it is reasonable to compare the results of the different data processing methods. Additionally, expiratory CO_2_-sampling was not performed, so the measured BOLD signal changes could not be normalized to CO_2_ changes and the actual vasodilatory stimulus achieved by each patient. We used the DSC MR data to correct for different blood arrival times in the brain, as recently proposed [8]. It is important to note that DSC MRI does not fully reflect BOLD lag. On the one hand, a gadolinium-based contrast agent does not show the same kinetic behavior as blood. On the other hand, temporal variations in the BOLD response are expected to be the sum of the variability in CVR response onset and the regional differences in blood transit time [23,26,68]. With DSC MRI, we only corrected for blood transit time, rather than delay in vasomotor response, which might be possible with alternative methods such as combined hypercapnic and hyperoxic gas inhalation [28] or with iterative data processing approaches during fMRI with CO_2_ inhalation [68]. Our approach of implementing a hypercapnia-triggered fMRI setup that could be implemented as easily as possible meant that we were unable to estimate the vasomotor response delay. To ensure optimal comparability with the [^15^O]water PET data, we used the same color scale for bh-fMRI maps of all patients. However, even among healthy individuals, the same breath-hold periods do not necessarily result in identical changes in CO_2_ [21,69], so differences in the BOLD signal changes are expected. Therefore, the color-coded scales might not have been optimal for all patients, which could have affected the rating. However, it should be emphasized that we focused particularly on side differences and negative CVR values in the rating because these criteria are clinically relevant and are unaffected by color coding.

The bh-fMRI technique has inherent limitations, such as the need for patient compliance [8,21,41]. To address this limitation, we used the cerebellar signal time course to verify patients’ compliance, as proposed in other studies [2,4,5,8].

In line with previous studies, this study further emphasizes the strong concordance between bh-fMRI and the reference standard [^15^O]water PET [2,4,8], even when standardized color scales are used. Further research should focus on optimizing the bh-fMRI technique and include, for example, expiratory CO_2_ sampling for optimal inter-personal comparability.

## 5. Conclusions

The main finding of this study was that analyzing single time points or short time periods of BOLD signal changes leads to the highest visual agreement between bh-fMRI CVR and [^15^O]water PET CPR maps in patients with MMA. This indicates that single time points or short time periods should be analyzed in routine clinical bh-fMRI scans of patients with MMA. Future studies should validate the findings with a VOI-by-VOI correlation analysis to the diagnostic standard in other diseases.

## Figures and Tables

**Table 1:** Summary of patient data.

Number of patients	47 ^2^
Age (median, range)	39, 14–64
Female:Male ratio	1.5:1
Days between MRI and PET data acquisition (median, range)	65, 1–168
Data sets with time period of 0–3 months between MRI and PET data acquisition	33
Data sets with time period of 3–6 months between MRI and PET data acquisition	13
Number of included data sets	46
Data sets without revascularization	44
Data sets after revascularization	2 (unilateral STA-MCA in both cases) ^1^

^1^ STA = superficial temporal artery, MCA = middle cerebral artery; ^2^ 47 patients met the inclusion criteria; data sets of 46 patients were evaluated, as one patient performed the breath-hold task incorrectly.

## Data Availability

In order to safeguard the confidentiality of the participants, the data pertaining to this study are currently withheld from public access. The data can be shared upon request.

## Supplementary Materials

The following supporting information can be downloaded at: https://www.mdpi.com/article/10.3390/diagnostics16060904/s1, Figure S1: Subgroup analysis of time periods of 0–3 months (top) and 3–6 months (bottom) between breath-hold fMRI (bh-fMRI) and [^15^O]water PET scan. The consensus ratings of the agreement between the bh-fMRI and the [^15^O]water PET maps are shown when evaluating bh-fMRI time periods of ±0 s, ±1 s, ±2 s, ±3 s, ±4 s, and ±5 s around the time point of the maximum cerebellar BOLD signal peak. Significant differences are marked on the right.

## Abbreviations

The following abbreviations are used in this manuscript:

ACZ	Acetazolamide
bh-fMRI	Breath-hold functional MRI
BOLD	Blood-Oxygenation-Level-Dependent
CPR	Cerebral perfusion reserve capacity
CVR	Cerebrovascular reactivity
DSC	Dynamic susceptibility contrast
EPI	Echo-planar imaging
FDR	False discovery rate
FWHM	Full width at half maximum
GLM	General linear model
MCA	Middle cerebral artery
MNI	Montreal Neurological Institute
MMA	Moyamoya Angiopathy
STA	Superficial temporal artery
TP_cereb. max_	Time point of the maximum cerebellar signal peak
