# Supplementary figures and images for: Cerebrovascular Reactivity Assessment with Breath-Hold Functional MRI in Patients with Moyamoya Angiopathy: Which Time Period to Analyze?
Source: Diagnostics (Basel). 2026 Mar 18;16(6):904. doi: 10.3390/diagnostics16060904 (PMC13025066; doi:10.3390/diagnostics16060904)

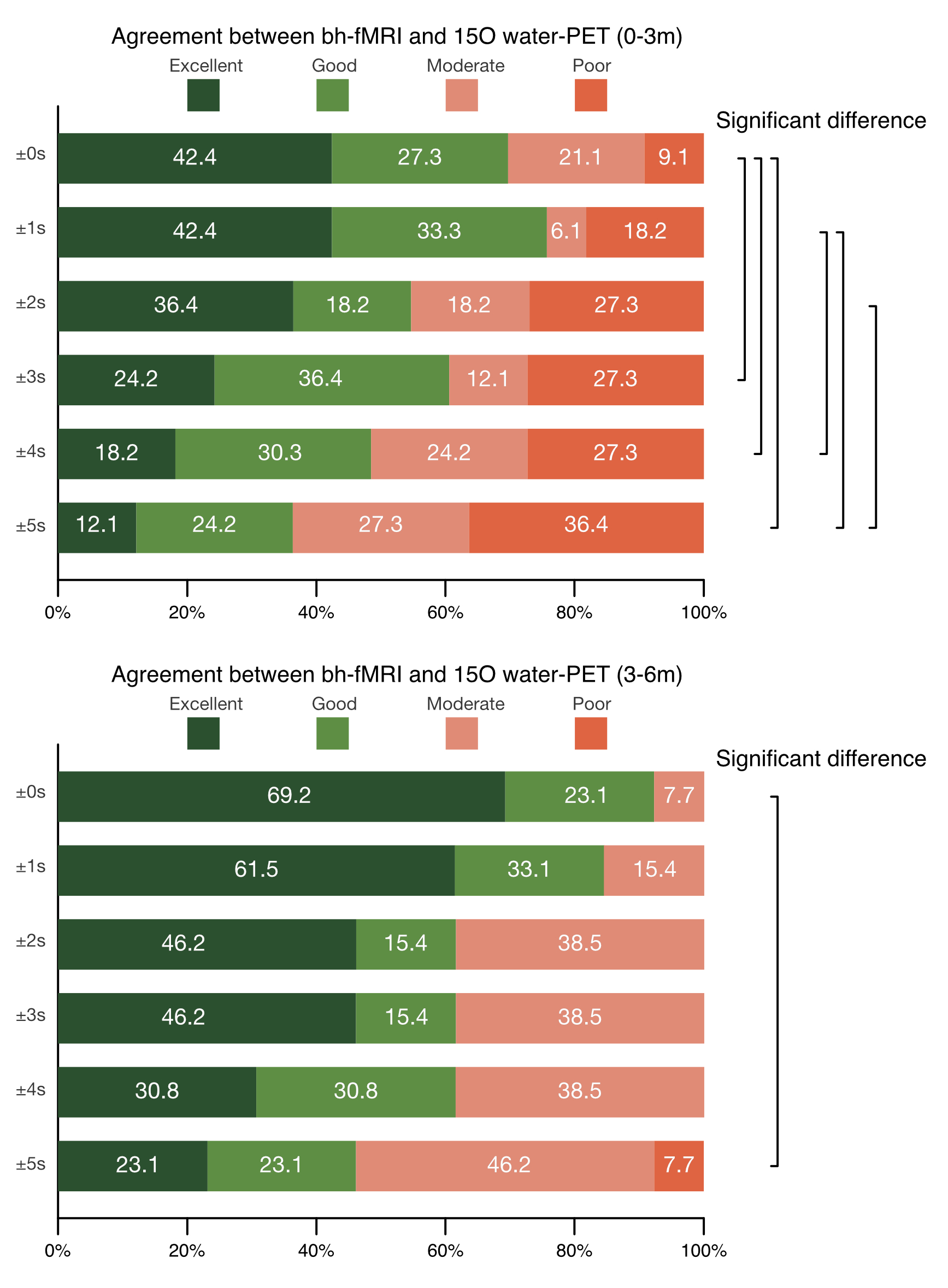

Supplement: Supplementary file 1 [file diagnostics-16-00904-s001.zip › FigureS1.jpg]
